# Microcredit participation and women’s health: results from a cross-sectional study in Peru

**DOI:** 10.1186/s12939-015-0194-7

**Published:** 2015-08-05

**Authors:** Rita Hamad, Lia C. H. Fernald

**Affiliations:** Division of General Medical Disciplines, Stanford University, 1070 Arastradero Road, Palo Alto, CA 94304 USA; School of Public Health, University of California Berkeley, 50 University Hall, Berkeley, CA 94720 USA

**Keywords:** Microcredit, Poverty alleviation, Women’s health, Socioeconomic determinants of health, Latin America, Peru

## Abstract

**Introduction:**

Social and economic conditions are powerful determinants of women’s health status. Microcredit, which involves the provision of small loans to low-income women in the hopes of improving their living conditions, is an increasingly popular intervention to improve women’s socioeconomic status. Studies examining the health effects of microcredit programs have had mixed results.

**Methods:**

We conduct a cross-sectional study among female clients of a non-profit microcredit program in Peru (*N* = 1,593). The predictor variable is length of microcredit participation. We conduct bivariate and multivariate linear regressions to examine the associations between length of microcredit participation and a variety of measures of women’s health. We control for participants’ sociodemographic characteristics.

**Results:**

We find that longer participation is associated with decreased depressive symptoms, increased social support, and increased perceived control, but these differences are attenuated with the inclusion of covariates. We find no association between length of participation and contraception use, cancer screening, or self-reported days sick.

**Conclusions:**

These results demonstrate a positive association between length of microcredit participation and measures of women’s psychological health, but not physical health. These findings contribute to the discussion on the potential of microcredit programs to address the socioeconomic determinants of health, and suggest that addressing socioeconomic status may be a key way to improve women’s health worldwide.

## Introduction

Social and economic conditions are powerful determinants of women’s health status. In low- and middle-income countries (LMIC), women with higher incomes, higher educational attainment, and greater levels of empowerment are more likely to seek out preventive health care, more apt to use modern contraceptive methods, and more likely to attend prenatal care visits [1, 2]. Policymakers have called for interventions to improve socioeconomic status as a means to address the fundamental determinants of health disparities among women [3].

One increasing popular tool to achieve these aims is microcredit [4]. This intervention involves the provisi on of small loans to low-income individuals – most often women – who are too poor to access traditional financial services, in the hopes that they will invest these funds in microenterprises to improve their families’ health and living conditions. There are currently over 3,000 organizations providing microcredit loans worldwide [5].

There are several pathways through which microcredit could improve health outcomes. If investments lead to increases in income, a family may have a greater ability to pay for food and other household resources, in addition to decreasing the stress associated with living in poverty [6]. Increased economic independence for women may enable them to be more active in decision-making around finances and health, thereby increasing their relative status within the household [7]. This may especially be true in microcredit programs in which women receive social support through participation in group lending models [8]. Some researchers have suggested that microcredit may be an effective tool to attain the Millennium Development Goals, international targets set by the United Nations and other global actors to improve poverty, education, and women’s empowerment [9].

There are reasons to be concerned that microcredit’s impacts may not be uniformly positive. Given that microcredit organizations provide loans rather than monetary transfers, participation may lead to increased debt and stress if the loans are not invested appropriately. For example, prior research has found that women may become trapped in a cycle of debt as they take out additional loans to repay old ones [10]. Moreover, women often manage the microenterprise in addition to running the household, resulting in a dual burden of responsibility and contributing to role overload or role conflict [11]. It is also likely that the structural contexts in which women live play a determining factor in the effects of microcredit programs on women’s health [12]. For instance, prior work has found that women in some microcredit programs are subject to domestic violence, and are often forced to take out loans by their husbands or other relatives [10].

Indeed, the impact of microcredit programs is controversial, with mixed findings based on recent systematic reviews [13, 14]. A major challenge has been the difficulty in conducting randomized controlled trials (RCTs), given the high penetration of microcredit interventions in LMIC that may lead to higher take-up in control groups and small or null effects. One RCT in Ethiopia randomized a group lending intervention among administrative areas, finding no change in economic or health outcomes among those communities randomized to the microcredit intervention [15]. Another study in India examined a variety of health and economic outcomes and found only an increase in spending on durable goods in the treatment group [16]. Another RCT demonstrated increased social capital among recipients, although the loans were combined with a “participatory gender training” and may not reflect the effects of the financial intervention itself [17]. Only one study to our knowledge has conducted a randomized experiment in an area previously devoid of microcredit – in Morocco – and this found no effects on economic or health outcomes in villages randomized to receive the intervention [18]. Given the unique circumstances in each cultural context, especially surrounding gender issues, the results of these studies may not be generalizable to other international settings.

There is a larger but still limited body of research examining the effects of microcredit on health outcomes using cross-sectional designs. These too demonstrate inconsistent findings. Studies have found conflicting associations of microcredit with women’s nutrition [19–21], empowerment [22, 23], contraceptive use [24–26], and mental health [27, 28]. Many of these studies suffer from selection bias, as they compare program participants with non-participants who have been shown to differ in ways that make them less likely to succeed than loan recipients [29].

The study described here adds to the literature on the associations between microcredit participation and health outcomes. We analyze the association between length of participation in a microcredit program among loan recipients in Peru using a cross-sectional design, examining a robust set of women’s health outcomes, including general health, mental health, and reproductive health. We hypothesize that longer participation in the microcredit program is associated with more favorable women’s health outcomes. In doing so, we provide evidence on whether an intervention designed to address women’s poverty in low-resource settings is associated with better health status.

## Methods

### Study design

**Fig. 1:**
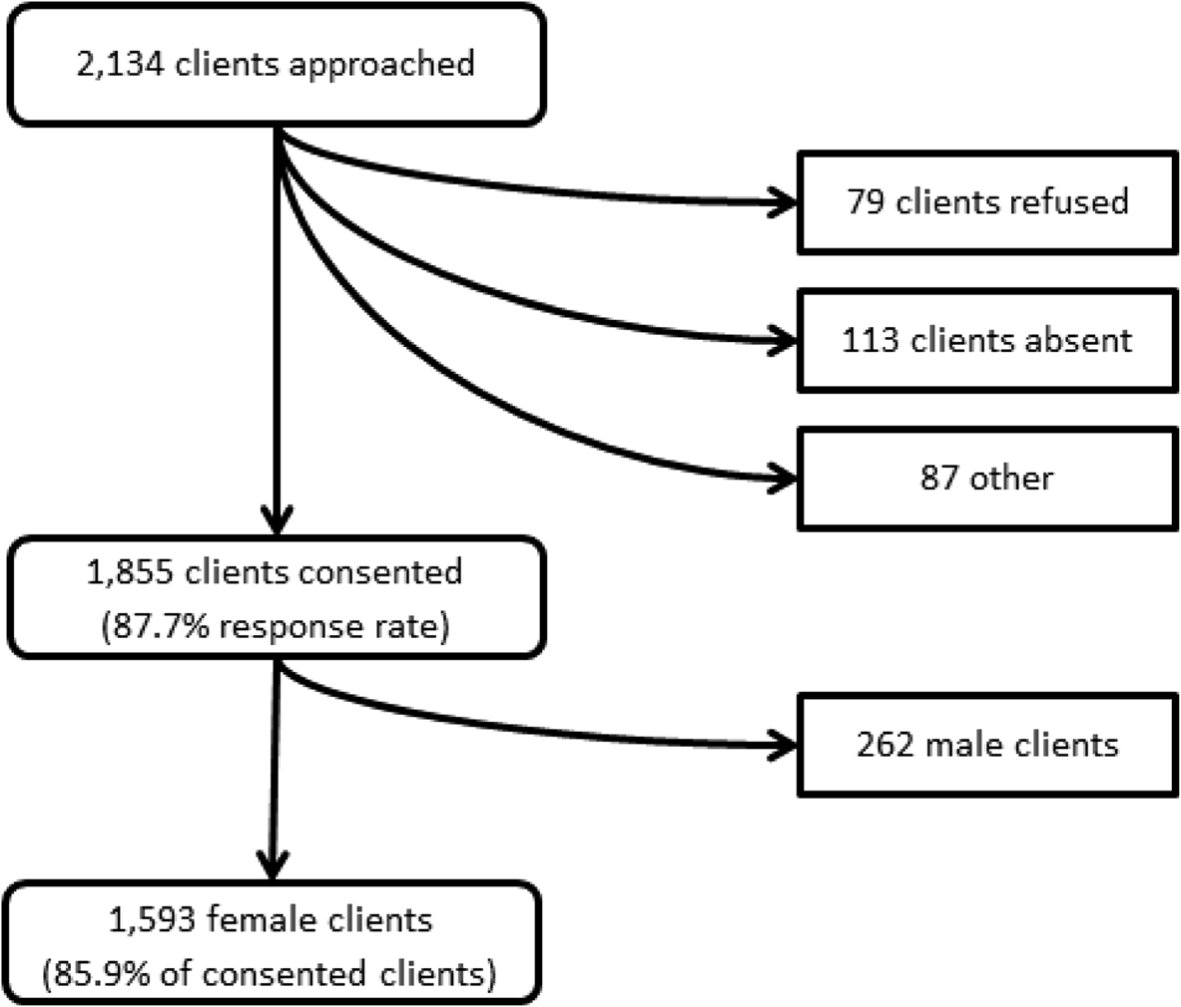
Sample framework

Data for this study were collected in February 2007 among participants in a microcredit program in Peru. We partnered with Prisma, a non-profit organization that provides microcredit and other social services throughout the country. We approached all the organization’s clients in Pucallpa (*N* = 2,134), a large city in the jungle (*selva*) region (population 136,000, 93 % urban). Of clients we approached, 1,855 agreed to participate in the survey (Fig. 1). The main reasons for non-response were refusal to participate and the client not being available for interview. No other information was available on non-responders. As prior studies have found differences in health effects of microcredit programs by client gender [28, 30], and because in this study we are interested in pathways that are specific to female clients, only female participants were included (*N* = 1,593).

Native Spanish speakers were hired and trained locally to conduct the surveys. The organization’s staff confirmed that all clients were comfortable communicating in Spanish and that organizational activities were conducted only in Spanish. The questionnaire, described below, was translated into Spanish by native speakers fluent in both English and Spanish. Focus groups were conducted among a small subset of clients to ensure question intelligibility, and validity testing was conducted to ensure intra- and inter-rater reliability. Clients were approached at their monthly meetings and invited to participate in the survey. Those who were absent from the meetings were telephoned or approached at home to schedule an interview appointment.

### Description of intervention

Clients in the sample were organized into 137 loan groups, each consisting of 10 to 20 individuals. These groups met on a monthly basis with a loan officer from the organization in order to make payments and conduct other administrative business. The average loan size in this sample was US$357, repaid over the course of a 6-month loan cycle, with a monthly interest rate of 4 %. This interest rate is similar to those among microcredit organizations in other LMIC [31]. Members were also required to deposit 2 % of the amount borrowed into a savings account with the organization.

New applicants were approved by the organization and by other members. This process allows poor individuals to provide social collateral for one another in the absence of physical collateral, a common structure in microcredit organizations [32].

Prisma is a non-profit organization that provides a variety of services to the urban and rural poor throughout Peru, including business training, rural development, and health education. At the time of the survey, the clients in this study were only receiving microcredit services.

### Measures

The primary predictor variable used in this study was length of participation in the microcredit program (range: 0 to 11 cycles). Clients were asked about the number of loan cycles they had completed with the organization (1 loan cycle = 6 months). Using length of participation as the independent variable is a method that has been used in prior studies to capture the “dose effect” of microcredit on a variety of outcomes [20, 24, 33, 34]. Importantly, longer participation may reflect the fact that women are benefiting from the intervention and choosing to continue, or it may be a result of being caught in a cycle of debt; we are not able to distinguish between these. We chose not to include non-clients as a control group, as these individuals may differ from clients in unobserved ways such as motivation or sense of entrepreneurship [35].

In the first set of models, number of loan cycles was included as a continuous variable. To capture potentially non-linear associations with the outcome variables, we also conducted a second set of analyses in which the number of completed loan cycles was transformed into a categorical variable: less than 3 loan cycles (1–17 months), 3–5 loan cycles (18-35 months), and 6 or more loan cycles (36+ months).

Health outcomes included a variety of indicators of women’s well-being. To capture general health, we asked women about the number of days they had been sick during the past month. Women indicated whether they had received a routine preventive cancer screening (e.g., Pap smear or mammogram) in the past year, a measure used previously to measure access to healthcare [36, 37]. Reproductive health was assessed by asking whether women discussed contraception with their partner, and whether they used any form of contraceptive method. Mental health was measured using the 20-item Center for Epidemiologic Studies – Depression scale (CES-D, range 0–60), which has been validated in low-income Spanish-speaking populations in the United States and Latin America [38, 39]. A score above 16 indicates a high risk for clinical depression in a US population [40]. Higher cut-off scores have been proposed in other populations, e.g., 35 based on a study conducted in a population of adult Mexican women [41, 42]. Social support was measured using the 11-item Duke-UNC Functional Social Support Questionnaire (range 0–55) [43], whose validity and reliability have been demonstrated in prior studies including Spanish-speaking populations [43, 44]. Finally, women responded to two questions about their perceived control over life circumstances and happiness with the level of control over life circumstances (range 0–6), similar to standard questions included in prior studies linking perceived control with socioeconomic status and other life stressors [45, 46]. Perceived control was included based on prevailing hypotheses that microcredit improves women’s health in part due to heightened empowerment and control over life circumstances [6], and based on longstanding evidence that perceived control mediates the pathway between material deprivation and health [47].

Covariates included women’s age, marital status (married or cohabitating vs. single), and a categorical variable representing educational attainment (primary or less, some secondary, complete secondary, at least some post-secondary). To flexibly model age, we included age-squared in regressions. To determine relative poverty, clients were asked about household assets, such as cars, refrigerators, and televisions. Information was also obtained about whether the client owned their home, and the materials from which the roof, walls, and floor were constructed. Based on the responses to these questions, principal components analysis was used to construct two continuous indices of household poverty: (1) assets, which included variables representing ownership of televisions, cars, blenders, refrigerators, radios, CD and DVD players, washing machines, fans, motorcycles, bicycles, and tractors; and (2) housing, which included variables representing home ownership and the materials from which the roof, walls, and floor were constructed. These questions were adapted from the Demographic and Health Surveys [48]. This technique has been used previously to measure the relative poverty of households in LMIC [49, 50]. Data on income were not available.

### Data analysis

We conducted multivariable linear regressions, using length of participation as a predictor to capture the dose effect of the microcredit program on women’s health. Linear probability models were used for binary dependent variables. We first conducted unadjusted analyses to examine the association between length of participation and women’s health outcomes. For those outcome variables with a statistically significant relationship with length of participation in unadjusted analyses, we then controlled for the sociodemographic characteristics outlined above. Robust standard errors were clustered at the level of the loan group. The number of observations differed across regressions due to client refusal or out-of-range responses.

Data were double-entered using CSPro 3.3 (U.S. Census Bureau, Population Division, Washington, D.C.). Statistical analyses were conducted using Stata 13 (Stata Corporation, College Station, TX).

### Ethics approval

The Institutional Review Boards of the University of California Berkeley and Prisma provided ethics approval for this study. Clients provided written or verbal consent for participation.

## Results

### Client characteristics

**Fig. 2:**
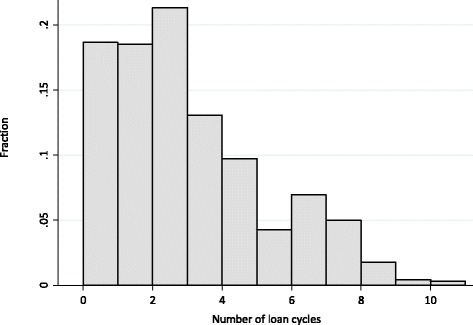
Distribution of number of loan cycles

Women in this sample (*N* = 1,593) were 39.5 years old on average and diverse with respect to educational attainment; about 80 % were married or co-habiting (Table 1). On average, women had completed 2.6 loan cycles (range 0 to 11, Fig. 2).

**Table 1:** Sample characteristics (*N* = 1,593)

Sociodemographic characteristics	
Age, years (mean ± SD)	39.5 ± 9.9
Education (%)	
Primary or less	27.0
Some secondary	27.2
Complete secondary	25.8
At least some post-secondary	20.0
Married or co-habiting (%)	80.2
Assets^a^ (mean ± SD)	0.055 ± 1.7
Housing^b^ (mean ± SD)	0.050 ± 1.5
Loan-related Characteristics	
Number of loan cycles completed	
Mean ± SD	2.6 ± 2.2
Median (interquartile range)	2 (1, 4)
Loan size (US$)	
Mean ± SD	354.3 ± 195.6
Median (interquartile range)	310.3 (206.9, 413.8)
Mental & Physical Health Characteristics	
Depressive symptoms^c^ (mean ± SD)	18.2 ± 8.7
Perceived social support^d^ (mean ± SD)	39.4 ± 8.3
Perceived control^e^ (mean ± SD)	7.4 ± 1.0
Discusses contraception with partner (%)	74.5
Uses contraception (%)	48.2
Days sick in last month (mean ± SD)	2.4 ± 6.1
Cancer screening in past year (%)	35.9

Note: Only female subjects included

^a^Assets variable constructed using principal components analysis. Assets included televisions, cars, refrigerators, fridge, radio, CD players, DVD players, washing machines, blenders, fans, motorcycles, bicycles, and tractors

^b^Housing variable constructed using principal components analysis. Elements included home ownership and construction materials for the roof, walls, and floor

^c^Assessed using the Center for Epidemiologic Studies Depression scale (range 0–60)

^d^Perceived social support measured using 11-item Duke-UNC Functional Social Support Questionnaire (range 0–55)

^e^Perceived control measured using a 2-item scale (range 0–6)

The average score for perceived depressive symptoms was 18.2, which is above the cut-off of 16 at which individuals are considered to be at increased risk of depression in the United States. About three-quarters of women reported discussing contraception with their partner, and less than half reported using any contraceptive method. Women reported an average of 2.4 sick days in the last month. About one-third reported receiving a routine cancer screening in the last year.

### Associations between microcredit participation and health

We first examined the associations between length of participation and health, using number of loan cycles as a continuous variable (Table 2). In unadjusted analyses, length of participation was significantly associated with decreased depressive symptoms (β = -0.29, *p* = 0.006), increased perceived social support (β = 0.25, *p* = 0.01), increased perceived control (β = 0.045, *p* < 0.001), and decreased contraception use (β = -0.015, *p* = 0.008), with a non-significant tendency towards increased discussion of contraception (β = 0.0080, *p* = 0.09).

**Table 2:** Unadjusted associations between length of microcredit participation (as continuous variable) and women’s health

	Marginal effect [95 % CI]						
	Depressive symptoms^a^	Social support^b^	Perceived control^c^	Discuss contraception	Use contraception	Cancer screening in last year	Days sick in last month
	*N* = 1,475	*N* = 1,547	*N* = 1,579	*N* = 1,449	*N* = 1,559	*N* = 1,507	*N* = 1,580
No. loan cycles	−0.29**	0.25*	0.045**	0.0080+	−0.015**	0.0089	0.12
	[−0.49, −0.08]	[0.054, 0.45]	[0.021, 0.069]	[−0.018, 0.0015]	[−0.026, −0.0039]	[−0.0027, 0.021]	[−0.031, 0.27]

Note: Unadjusted ordinary least squares regression is used for continuous outcomes and linear probability models are used for binary outcomes. Standard errors are clustered by loan group

*p < 0.05, + *p* < 0.1, **p < 0.01

^a^Assessed using the Center for Epidemiologic Studies Depression scale (range 0–60)

^b^Perceived social support measured using 11-item Duke-UNC Functional Social Support Questionnaire (range 0–55)

^c^Perceived control measured using a 2-item scale (range 0–6)

For outcomes with statistically significant associations in unadjusted analyses, we then controlled for sociodemographic factors including age, marital status, educational attainment, and indices for assets and housing (Table 3). In these models, longer program participation remained significantly associated with greater perceived control (β = 0.037, *p* = 0.002); it also had a non-significant tendency to be associated with reduced depressive symptoms (β = −0.20, *p* = 0.06) and greater perceived social support (β = 0.19, *p* = 0.08). Program participation was not significantly associated with reproductive health, cancer screening, or days sick, perhaps due to the significant association of age and marital status with length of participation.

**Table 3:** Adjusted associations between length of microcredit participation (as continuous variable) and women’s health

	Marginal effect [95 % CI]				
	Depressive symptoms^a^	Social support^b^	Perceived control^c^	Discuss contraception	Use contraception
	*N* = 1,445	*N* = 1,516	*N* = 1,548	*N* = 1,426	*N* = 1,528
No. loan cycles	−0.20+	0.19+	0.037**	0.00043	0.0014
	[−0.42, 0.0089]	[−0.021, 0.40]	[0.013, 0.061]	[−0.008, 0.0096]	[−0.0091, 0.012]
Age	0.055	−0.16	0.024	0.024**	0.015+
	[−0.26, 0.37]	[−0.44, 0.13]	[−0.015, 0.063]	[0.011, 0.038]	[−0.00061, 0.031]
Age-squared	−0.00098	0.0021	−0.00022	−0.00041**	−0.00039**
	[−0.0049, 0.0029]	[−0.0014, 0.0056]	[−0.00068, 0.00024]	[−0.00059, −0.00024]	[−0.00057, −0.00022]
Married	−0.53	0.26	−0.19**	0.30**	0.21**
	[−1.74, 0.69]	[−0.87, 1.39]	[−0.30, −0.086]	[0.22, 0.38]	[0.16, 0.27]
Education (ref: less than secondary)					
Some secondary	−1.42*	0.21	0.024	0.031	0.037
	[−2.81, −0.028]	[−0.89, 1.31]	[−0.11, 0.16]	[−0.036, 0.097]	[−0.037, 0.11]
Complete secondary	−2.22**	2.12**	0.22**	0.090**	0.030
	[−3.57, −0.87]	[1.11, 3.13]	[0.060, 0.37]	[0.029, 0.15]	[−0.038, 0.099]
Post-secondary	−3.21**	3.20**	0.16+	0.043	0.046
	[−4.89, −1.53]	[1.83, 4.56]	[−0.012, 0.34]	[−0.028, 0.11]	[−0.039, 0.13]
Assets^d^	−0.46**	0.17	0.015	0.014+	0.0058
	[−0.77, −0.15]	[−0.12, 0.45]	[−0.021, 0.051]	[−0.00079, 0.029]	[−0.0094, 0.021]
Housing^e^	0.15	0.31	0.037+	0.012	−0.0077
	[−0.30, 0.59]	[−0.092, 0.71]	[−0.0070, 0.0805]	[−0.0091, 0.034]	[−0.031, 0.016]
Constant	20.12**	40.21**	6.74**	0.17	0.33+
	[13.88, 26.36]	[34.76, 45.65]	[5.90, 7.58]	[−0.13, 0.46]	[−0.011, 0.68]

Ordinary least squares regression is used for continuous outcomes and linear probability models are used for binary outcomes. Standard errors are clustered by loan group

*p < 0.05, + *p* < 0.1, **p < 0.01

^a^Assessed using the Center for Epidemiologic Studies Depression scale (range 0–60)

^b^Perceived social support measured using 11-item Duke-UNC Functional Social Support Questionnaire (range 0–55)

^c^Perceived control measured using a 2-item scale (range 0–6)

^d^Assets variable constructed using principal components analysis. Assets included televisions, cars, refrigerators, fridge, radio, CD players, DVD players, washing machines, blenders, fans, motorcycles, bicycles, and tractors

^e^Housing variable constructed using principal components analysis. Elements included home ownership and construction materials for the roof, walls, and floor

### Length of participation as a categorical variable

We next examined whether there were non-linear associations between length of participation and health by transforming number of loan cycles into a categorical variable (Table 4). The association between number of loan cycles and lower depressive symptoms was significant for those who had participated for 6 or more loan cycles when compared with those who had participated for less than 3 cycles (β = 1.45, *p* = 0.04), but not for those who had participated for 3–5 loan cycles as compared to less than 3 cycles (β = 0.95, *p* = 0.08). Perceived control was greater in those who had participated for 3–5 loan cycles as compared to less than 3 loan cycles (β = 0.13, *p* = 0.03), and greater still for those who had completed 6 or more loan cycles compared with less than 3 loan cycles (β = 0.19, *p* = 0.02). There was also a non-significant tendency towards improved social support among those with 3–5 cycles of participation compared to less than 3 cycles (β = 0.95, *p* = 0.08). As with the analyses described above, there were no significant associations for reproductive health, cancer screening, or days sick.

**Table 4:** Associations between length of microcredit participation (as categorical variable) and women’s health

	Marginal effect [95 % CI]				
	Depressive symptoms^a^	Social support^b^	Perceived control^c^	Discuss contraception	Use contraception
	*N* = 1,445	*N* = 1,516	*N* = 1,548	*N* = 1,426	*N* = 1,528
No. loan cycles (ref <3)					
3–5	0.16	0.95+	0.13*	0.018	−0.0028
	[−0.87, 1.20]	[−0.12, 2.03]	[0.016, 0.25]	[−0.036, 0.072]	[−0.058, 0.052]
6+	−1.45*	0.70	0.19*	0.020	0.028
	[−2.80, −0.11]	[−0.71, 2.12]	[0.029, 0.35]	[−0.042, 0.082]	[−0.039, 0.095]
Age	0.048	−0.16	0.024	0.024**	0.015+
	[−0.27, 0.36]	[−0.44, 0.13]	[−0.015, 0.063]	[0.010, 0.038]	[−0.00054, 0.031]
Age-squared	−0.00089	0.0021	−0.00022	−0.00042**	−0.00040**
	[−0.0047, 0.0030]	[−0.0014, 0.0057]	[−0.00068, 0.00023]	[−0.00059, −0.00024]	[−0.00057, −0.00022]
Married	−0.52	0.27	−0.19**	0.30**	0.21**
	[−1.74, 0.70]	[−0.86, 1.39]	[−0.298, −0.086]	[0.22, 0.38]	[0.16, 0.27]
Education (ref: less than secondary)					
Some secondary	−1.33+	0.26	0.025	0.032	0.035
	[−2.72, 0.054]	[−0.86, 1.37]	[−0.11, 0.16]	[−0.035, 0.099]	[−0.038, 0.11]
Complete secondary	−2.18**	2.14**	0.21**	0.090**	0.030
	[−3.53, −0.83]	[1.13, 3.16]	[0.060, 0.37]	[0.029, 0.15]	[−0.039, 0.098]
Post-secondary	−3.15**	3.25**	0.17+	0.044	0.045
	[−4.83, −1.47]	[1.88, 4.63]	[−0.0076, 0.34]	[−0.028, 0.12]	[−0.039, 0.13]
Assets^d^	−0.47**	0.17	0.015	0.013+	0.0054
	[−0.78, −0.16]	[−0.11, 0.45]	[−0.022, 0.051]	[−0.0014, 0.028]	[−0.0098, 0.021]
Housing^e^	0.16	0.32	0.037+	0.012	−0.0082
	[−0.28, 0.60]	[−0.090, 0.72]	[−0.0071, 0.081]	[−0.0095, 0.033]	[−0.032, 0.016]
Constant	19.85**	40.21**	6.75**	0.16	0.34+
	[13.58, 26.12]	[34.71, 45.71]	[5.92, 7.58]	[−0.13, 0.46]	[−0.0086, 0.68]

Ordinary least squares regression is used for continuous outcomes and linear probability models are used for binary outcomes. Standard errors are clustered by loan group

*p < 0.05, + *p* < 0.1, **p < 0.01

^a^Assessed using the Center for Epidemiologic Studies Depression scale (range 0–60)

^b^Perceived social support measured using 11-item Duke-UNC Functional Social Support Questionnaire (range 0–55)

^c^Perceived control measured using a 2-item scale (range 0–6)

^d^Assets variable constructed using principal components analysis. Assets included televisions, cars, refrigerators, fridge, radio, CD players, DVD players, washing machines, blenders, fans, motorcycles, bicycles, and tractors

^e^Housing variable constructed using principal components analysis. Elements included home ownership and construction materials for the roof, walls, and floor

## Discussion

This study adds to the literature on health outcomes among clients of microcredit programs by examining a variety of indicators of women’s health status among clients of a non-profit organization in Peru. We find that longer participation is associated with better psychological outcomes – including depressive symptoms, perceived social support, and perceived control – but not associated with general health and reproductive health outcomes. Transforming number of loan cycles into a categorical variable demonstrates the non-linear nature of this relationship, in that decreased depressive symptoms, increased perceived control, and greater perceived social support (non-significant trend) are more pronounced among those with longer program participation as compared to those with shorter length of participation. These findings suggest that there are cumulative positive effects associated with longer participation, although the cross-sectional design precludes us from determining whether this association is causal.

The null associations for other health outcomes suggest that microcredit may not be effective in improving women’s physical health in the absence of other structural interventions to their communities, such as healthcare access or transportation. Possible solutions include the provision of specific synergistic interventions that use microcredit programs as a platform for the provision of health services. Alternatively, this may speak to the limitation of microcredit as an intervention to improve women’s health, and the need for other infrastructure changes implemented by community organizations and governments.

The mental health associations we demonstrate are consistent with a prior study in Bangladesh that found that microcredit participants experience less stress than non-participants [33], and others that have found greater women’s empowerment among microcredit clients [23, 51]. These findings may be due to the increased income available to these women, or to the increased opportunities for networking in the context of loan groups [6]. Other studies, however, have found worsened stress and depressive symptoms among female clients. Several qualitative studies have suggested that tense household dynamics and increased debt burden may be contributing to worsened mental health among female clients [11, 22], but it may be that relations among members at Prisma are more supportive. The findings highlight the importance of conducting evaluations across country settings since community contexts may differ in important ways. Of note, we find that the average score for depressive symptoms among participants is above the U.S. cut-off of 16 that indicates a high risk for clinical depression. While this seems high, it is similar to other vulnerable populations in prior studies in Peru [52, 53]. It is possible that a higher cut-off for the CES-D is appropriate in this population, and this can be explored in future studies.

We find no association between length of program participation and discussion or use of contraceptive methods, although prior studies have suggested that women discuss and evaluate contraceptive methods in the context of their social networks [54, 55], and that women’s empowerment is associated with higher contraceptive use [56, 57]. Self-reported contraception use in this sample is similar to that among women in Peru [58]. Prior studies of microcredit programs have found positive, negative, and no effects on contraceptive use [24–26]. It may be that gender or household dynamics in this community constrain women’s ability to apply their increased perceived control to their reproductive health. Future studies could examine whether the women’s increase in perceived control is isolated to certain domains – e.g., finances or employment, and not health or relationships – as prior research has found that an individual’s perceived control may vary across domains [59].

The prevalence of cervical cancer screening in this sample is similar to findings from prior studies in Peru [60]. This study’s finding that microcredit participation was not associated with general health (as measured by days sick) or changes in cancer screening is consistent with prior studies in which participation in a microcredit program did not change prevalence of cervical cancer screening in the absence of an accompanying health promotion intervention [36, 37]. This contradicts findings that employed individuals and those with higher income are more likely to seek preventive healthcare [61–63]. It may be that other barriers to healthcare access – such as poor transportation or clinic availability – continue to limit women’s ability to obtain preventive healthcare. It may also be that this microcredit program may not have a significant impact on clients’ income, although this is not something that we can assess with this data set.

This study is limited in several ways. First, the study is cross-sectional in design, which precludes the ability to make causal inferences about the results. In other words, the positive associations for depressive symptoms, social support, and perceived control may be a result of confounding or reverse causality, in that those with better mental health are more likely to stay in the program. While we avoid bias caused by selection into the microcredit program by comparing existing clients with differing lengths of program participation, the results may suffer from survivorship bias in that those who remain clients differ from those who drop out. It is not clear in which direction this would bias the results, e.g., individuals may drop out because they graduated to a higher income level or because they failed to repay their loans. Compared to the general population in the jungle region, women in this sample are more educated and report greater ownership of assets and housing quality [64]. They are also more likely to be married or co-habiting. This may suggest possible survivorship bias. Yet even those clients in the sample who had most recently joined the organization – less than one year prior – demonstrated higher socioeconomic status than the general population in the jungle region based on (data not shown), suggesting that there is selection into the program. While the ideal solution would be to implement a randomized controlled trial, these are difficult to conduct in practice given the high penetration of microcredit programs in LMIC. One such study reports 27 % take-up in the treatment group compared to 18 % in the control group, resulting in reduced effect sizes [16]. Also, the study is limited in its use of length of participation as the predictor variable: longer participation is likely to represent continued benefit from the intervention and voluntary participation, although it may also reflect women getting caught in a cycle of debt. This may explain some of the null findings in our study for some outcomes. Future qualitative work could explore the nature of women’s decisions to continue participation in microcredit programs. Another limitation of this study is that it may not be representative of microcredit programs in other cultural contexts, as gender dynamics differ across and even within countries, and studies have found that the environment in which a microcredit intervention operates affects its ability to lift clients out of poverty [65, 66]. This study contributes to the literature on the implementation of these programs in a Latin American context, however, and future research should attempt to replicate these results in other settings.

## Conclusion

Addressing socioeconomic status may be a key way to improve women’s health worldwide. In this study we evaluate a microcredit intervention in Peru designed to alleviate poverty among low-income women. These results suggest that microcredit participation is associated with more favorable psychological measures among female clients. We did not find changes in general or reproductive health, however, suggesting that the potential impacts of microcredit programs may be limited in the absence of sociocultural or infrastructural improvements in surrounding communities. Future research could employ longitudinal or randomized methodologies to determine the causal role of microcredit in bringing about these changes, while qualitative studies could explore the ways in which programs can facilitate improvements in clients’ mental and physical health.

## Abbreviations

CES-D: Center for Epidemiologic Studies – Depression scale
LMIC: Low- and middle-income countries
PC: Principal components
RCT: Randomized controlled trial
